# Supplementary material for: Fast growth rate is associated with musculoskeletal biomechanical imbalance and dorsal cranial myopathy in broiler chickens
Source: PLoS One. 2025 Sep 18;20(9):e0332693. doi: 10.1371/journal.pone.0332693 (PMC12445488; doi:10.1371/journal.pone.0332693)
Supplement: Attachment — Submitted filename: Response to reviewers.pdf [file pone.0332693.s001.pdf]

Botucatu, August 6, 2025

Academic Editor Dr. Arda Yildirim

Dear Editor,

We are very happy to receive these valuable suggestions for improving our manuscript. We applied the corrections as suggested by the reviewers, and reviewed typos, clarified points that seemed unclear, and adjusted some other details as requested. Our detailed responses are shown in blue below. We look forward to receiving your decision.

Journal requirements:

1. Please ensure that your manuscript meets PLOS ONE's style requirements, including those for file naming. The PLOS ONE style templates can be found at

https://journals.plos.org/plosone/s/file?id=wjVg/PLOSOne_formatting_sample_main_body.pdf
and
https://journals.plos.org/plosone/s/file?id=ba62/PLOSOne_formatting_sample_title_authors_affiliations.pdf

Thank you for your suggestion. We double-checked our manuscript to ensure it meets PLOS ONE’s style requirements.

2. When completing the data availability statement of the submission form, you indicated that you will make your data available on acceptance. We strongly recommend all authors decide on a data sharing plan before acceptance, as the process can be lengthy and hold up publication timelines. Please note that, though access restrictions are acceptable now, your entire data will need to be made freely accessible if your manuscript is accepted for publication. This policy applies to all data except where public deposition would breach compliance with the protocol approved by your research ethics board. If you are unable to adhere to our open data policy, please kindly revise your statement to explain your reasoning and we will seek the editor's input on an exemption. Please be assured that, once you have provided your new statement, the assessment of your exemption will not hold up the peer review process.

Data underlying this manuscript will be made fully accessible through the Virginia Tech Data Repository upon acceptance of the manuscript. The dataset is already submitted to the repository and is just awaiting review and acceptance of the manuscript to generate a DOI. We are confident in the timely publication of the dataset through our institution.

3. Please upload a copy of Supporting Information Figure/Table/etc. which you refer to in your text on page 25.

Thank you for your comment. There is no supporting information (e.g. supplementary material) associated with this manuscript. All figures and tables are now available in the manuscript body.

Additional Editor Comments:

Dear Authors, Thank you for your submission. The reviewers found your study to be original and relevant, with potential value for both poultry science and broader animal welfare discussions. However, they also raised important points regarding the need for additional microscopic or molecular support, clarification of certain terms and procedures, and improved accessibility of supplementary materials. In light of these comments, a major revision is required before a final decision can be made. We encourage you to carefully address all reviewer suggestions to enhance the clarity and scientific rigor of your manuscript. Best regards, Arda Yıldırım

We appreciate your comments, and we have carefully addressed all reviewers' suggestions. In the present study, we did not conduct microscopic or molecular assessments of muscle tissue, as these analyses were not within the scope of this study's objectives. Regarding supplementary materials, there are no supporting files associated with this manuscript. All figures and tables are included within the main text. Therefore, we have removed the Supporting Information section from the manuscript.

Reviewer #1: In this manuscript, three different strains of broiler chicken were compared in terms of gait, posture and myopathies in the M. latissimus dorsi. The manuscript is very well written, the contents can well be understood also by readers not that much familiar with poultry science. The contents are of high relevance demonstrating that high and disproportionate growth is at the costs of animal welfare. Research on myopathies in broiler chickens is largely focused on the pectoralis muscle as the most important muscle in terms of the meat product and its high share of total muscle mass. However, other muscles balancing the body posture must also be considered, and the non-invasive photogrammetric method described herein provides a valuable tool for assessing and predicting imbalances in the musculo-skeletal system.

Thank you for your comments. We appreciate the time you took to make meaningful suggestions to improve the manuscript. We have responded to each comment in detail below.

Since PlosOne is targeting a broad scientific community, some poulty commons should be explained to non-expert readers: (1) the different times on the different diets (starter, grower, and finisher) were assumingly related to the growth velocity of the strains. However, it would be interesting to get to know whether the age at shifting to the next day was based on reaching a certain body weight and how the time of changing the diets might be related to some portion of the adult weight the animals would theoretically reach.

Thank you for your comment. We added some more detail as suggested: “Each diet was provided for approximately 1/3rd of the production period, which was dependent on growth rates.” (L141-143).

(2) Some conclusions or suggestion would be helpful on how to implement the findings about genetic strains into chicken meat production systems aiming to improve health and welfare of the birds. A hypercaloric diet was mentioned in Line 366, but this might need some discussion in terms of feed efficiency. What losses and what gains would be encountered if using slower growing strains? Would a change in feeding or in final slaughter weight help? Is the posture assessment potentially applicable under field conditions?

Thank you for your comments. We modified the conclusions as suggested:

- “Our results suggest that intermediate-growing strains offer a better balance between growth rate and leg health compared to slow-growing strains when fed diets as in this study.” (419-421).
- “Based on our findings, the intermediate-growing strain appears to be the most suitable for improving chicken health while maintaining reasonable growth rate under conventional broiler production systems with a target slaughter weight of 3.7 kg.” (L477-479).
- “This method can be used as a cost-effective indicator for monitoring musculoskeletal imbalance, dorsal cranial myopathy, and leg health in commercial settings.” (L483-485).

Unfortunately, our experimental design and findings do not allow us to fully address the reviewer’s questions regarding the effects of slowing-growing strains or changes in feeding strategies and final slaughter weight on economic parameters. However, we added a statement recommending future studies to assess these factors:

“We recommend further studies comparing performance and economic parameters of intermediate- and slow-growing broilers raised in conventional production systems to confirm our findings.” (L421-423).

Line 33: Correct typo in “Five bids/pen”.

Thank you for your suggestion, we modified as suggested: “Five birds/pen (n = 30 birds/genetic strain)” (L32).

Line 366: some explanation about the term “hypercaloric diet” should be provided (how much over needs, why used…).

Thank you for your comment. we modified as suggested:

- “The poor gait at 3.7 kg could be linked to the housing conditions and the diets used in this study that exceeded the recommended metabolizable energy (kcal/kg) requirements for

slow-growing broilers [42]. All strains were raised in pens in a poultry barn without outdoor access, relatively high stocking density, and received the same commercial diet to ensure consistent experimental conditions across all treatments." (L369-373).

- "Additionally, the *ad libitum* diets in this study were formulated for fast-growing broilers [25], providing 120-240 kcal/kg more than required by slower-growing strains [42]." (L378-380).

Reviewer #2: This is an interesting study that investigates dorsal cranial myopathy (DCM) through the lens of musculoskeletal biomechanical balance and gait analysis.

Thank you for your comments. We appreciate the time you took to make meaningful suggestions to improve the manuscript.

Major Comments:
The study would be significantly strengthened by the inclusion of microscopic findings or molecular/biomarker data to support the macroscopic observations. As it stands, the conclusions are based solely on gross morphological assessments, which limits the depth of pathological characterization.

Thank you for your valuable suggestion. We did not conduct microscopic or molecular assessments of muscle tissue, as these analyses were not within the scope of this study's objectives. These lesions can be macroscopically detected and are routinely done so during carcass grading at processing plants (for instance see https://doi.org/10.1016/j.japr.2021.100182 stating "The Official Inspection Service in Brazil performs the diagnosis of the DCM through visual inspection …") and in other studies besides our own (for instance see https://journals.pan.pl/Content/128555/PDF/17%20_%20Lebednikaite.pdf).

I was unable to access the supplementary materials submitted with the manuscript. Please ensure that these documents are available and accessible for review.

Thank you for your comment. There are no supporting documents (supplementary material) associated with this manuscript. All figures and table are available in the manuscript body. We misunderstood the PLOS ONE guidelines for Supporting Information. Therefore, we removed the supporting information section from the manuscript.

Minor Comments:
Line 188 – (P.374): It is unclear what this reference (P.374) pertains to. Please clarify or correct the citation.

Thank you for your comment. The citation "(p. 374)" was not intended as a separate reference but referred to the specific page in reference [19] where the following statement appears: "The sphere

radius was measured, and this value was constant for all analyzed images, as the birds had similar weight and shape." To improve clarity, we have revised the citation as follows:

"The sphere radius was measured, and this value was constant for all analyzed images, as the birds had similar weight and shape [19, p. 374]." (L191–192).
